# Chloroquine mitigates long-term effects of in vitro culture in mouse embryos 

**DOI:** 10.3389/fcell.2025.1640986

**Published:** 2025-08-12

**Authors:** Kaname Sato, Itsuki Koide, Md Wasim Bari, Satoshi Kishigami

**Affiliations:** ^1^ Department of Integrated Applied Life Science, Integrated Graduate School of Medicine, Engineering, and Agricultural Sciences, University of Yamanashi, Kofu, Japan; ^2^ Graduate School of Life and Environmental Sciences, Integrated Graduate School of Medicine, Engineering, and Agricultural Sciences, University of Yamanashi, Kofu, Japan; ^3^ Center for advanced Assisted Reproductive Technologies, University of Yamanashi, Kofu, Japan

**Keywords:** chloroquine, *in vitro* culture, mouse embryo, autophagy, DOHAD

## Abstract

**Background:**

*In vitro* culture of preimplantation embryos may increase the risk of long-term effects, such as obesity and metabolic diseases later in life in the offspring. While the long-term consequences of low-protein diets during early development have been reported in the context of DOHaD (Developmental Origins of Health and Disease) theory, the relationship between nutrient supply via autophagy during preimplantation development and these long-term effects remains unclear. In this study, we aimed to determine whether autophagy activity during *in vitro* culture of mouse embryos contributes to long-term effects, using chloroquine (CQ), a known autophagy inhibitor. Preimplantation embryos were cultured *in vitro* in the presence of CQ. The purpose was to investigate the long-term consequences of nutrient deprivation during preimplantation development under conditions of autophagy inhibition.

**Methods:**

Two-cell stage embryos were obtained by mating ICR female mice with ICR male mice, followed by oviduct flushing. The recovered embryos were cultured *in vitro* in CQ-supplemented medium. At the blastocyst stage, cultured embryos were immunostained with anti-Nanog and Cdx2 antibodies to assess blastocyst quality. Offspring derived from CQ-treated embryos were obtained by transferring the cultured embryos to pseudopregnant ICR females. At 8 weeks or later of age, offspring were examined using a glucose tolerance test.

**Results:**

We found that low concentration CQ significantly reduced developmental rate and total cell count in a CQ concentration-dependent manner (control: 67 ± 2.5 vs. 48 ± 2.3 with 1.0 µM CQ vs. 37 ± 2.9 with 2.0 µM CQ), as well as the numbers of trophectoderm (TE) and inner cell mass (ICM) cells. These results suggest that low concentration CQ treatment may suppress cell proliferation likely by inhibiting nutrient supply via autophagy. Notably, after implantation, the 2.0 µM CQ-treated group exhibited increased pups rate and reduced body weight comparable to the naturally mated group, and glucose tolerance similar to that of the naturally mated group, in contrasted to the untreated group.

**Discussion:**

These findings suggest that inhibiting autophagy during preimplantation development may mitigate the long-term effects of *in vitro* culture and support normal postnatal growth and metabolism. Thus, autophagy activity in early development may be a key cellular process underlying long term effects observed at later stages.

## 1 Introduction

The Developmental Origins of Health and Disease (DOHaD) theory proposes that the risk of developing lifestyle-related diseases such as diabetes and hypertension is strongly influenced by the health and nutritional environment during the fetal period and immediately after birth (Langley-Evans and McMullen, 2010; Aiken and Ozanne, 2014). In particular, fetal exposure to undernutrition during gestation—caused by external nutritional deficiency—can result in intrauterine growth restriction and epigenetic regulation of gene expression favoring nutrient conservation. These adaptations may predispose individuals to health issues later in life, including glucose intolerance and excessive growth in adulthood (Roseboom et al., 2006; Lehnen et al., 2013; Velazquez et al., 2019; Yura et al., 2005).

The preimplantation mammalian embryo is highly sensitive to its developmental environment, including factors such as nutrition, which can lead to long-term alterations in fetal and postnatal growth and phenotype (Velazquez et al., 2023). In the above context, maternal nutritional deficiencies—such as low protein intake during the preimplantation period—can influence gene expression and developmental programming, resulting in lasting changes in the offspring’s phenotype (Snoeck et al., 1990; Chamson-Reig et al., 2006; Calzada et al., 2016). These early nutritional disruptions have also been associated with an increased risk of cardiovascular and metabolic diseases in adulthood (Langley-Evans et al., 1996; Watkins et al., 2010).

During preimplantation development, nutrient supply from culture media and metabolism is considered important for long-term effects on embryonic development and postnatal development. *In vitro* culture exposes the preimplantation embryo to a non-physiological environment during embryonic culture, which has been linked to changes in embryonic growth and development in animal models and humans (Fleming et al., 2004). In addition, *in vitro* culture of preimplantation embryos can be a factor affecting birth weight (Dumoulin et al., 2010), the development of cardiovascular dysfunction in adults (Watkins and Fleming, 2009), abnormal placental morphology, placental DNA methylation levels (Vrooman, et al., 2022), and impaired glucose tolerance (Calle et al., 2012). However, the relationship between *in vitro* nutritional supply and the long-term effects of the nutritional environment on postnatal development remains unclear.

Autophagy is a major intracellular degradation and recycling mechanism that breaks down proteins and organelles. There are three main types of autophagy: macroautophagy, microautophagy, and chaperone-mediated autophagy (Vargas et al., 2023). Macroautophagy (hereafter referred to as autophagy) plays physiologically and pathologically important roles (Komatsu et al., 2005; Yang and Klionsky, 2009; Mijaljica et al., 2011; Morishita and Mizushima, 2019). A key function of autophagy is the formation of sequestration membranes in the cytoplasm-known as autophagosomes-in response to nutrient starvation, allowing cells to adapt by recycling intracellular components (Xie and Klionsky, 2007; He and Klionsky, 2009; Rabinowitz and White, 2010). Autophagosomes are double-membrane lipid bilayer structures that subsequently fuse with lysosomes, where their contents are degraded to release nutrients such as amino acids (Feng et al., 2014; Mehrpour et al., 2010). Other roles include maintaining intracellular homeostasis by removing unwanted intracellular proteins and damaged mitochondria, thereby preventing their accumulation (Mizushima, 2007). While it has been known for decades that autophagy, a conserved lysosomal degradation pathway, is highly active during differentiation and development, the functional significance of this activity was unknown until the discovery of autophagy-related (ATG) genes in the 1990s (Tsukada and Ohsumi, 1993). In recent years, the analysis of whole-body and tissue-specific knockout models of the ATG gene in mice has led to an explosion of knowledge regarding the function of autophagy in mammalian development and differentiation (Eskelinen et al., 2002; Kuma et al., 2004; Baerga et al., 2009).

Autophagy in preimplantation embryos is induced after fertilization and its expression is upregulated in early mouse embryos and that autophagy is an essential process in mammalian embryogenesis. Genetic studies in preimplantation mouse embryos have shown that autophagy is activated immediately after fertilization. Embryos deficient in autophagy, such as those lacking the autophagy-related gene Atg5, fail to develop beyond the 4- to 8-cell stage and undergo preimplantation lethality (Tsukamoto et al., 2008a). These findings indicate that autophagy is an essential process in mammalian embryogenesis. In response to nutrient starvation during the preimplantation period, autophagy may contribute to securing nutrients required for embryonic development and cell differentiation by actively degrading oocyte-derived proteins and cytoplasmic components. While this process reflects intracellular nutrient deprivation, the relationship between autophagy-mediated nutrient supply during this stage and its potential long-term effects remains unclear.

Chloroquine (CQ) is widely used as an anti-malarial drug (O’Neill et al., 1998; Al-Bari, 2015) and as a treatment for inflammatory diseases (Ferreira et al., 2021). It is also known as an autophagy inhibitor (Mauthe et al., 2018) and has been applied to preimplantation embryos from previous studies (Pasquier, 2015). In this study, we tested the hypothesis that nutrient supply or degradation mediated by autophagy during the preimplantation period influences long-term developmental outcomes. To investigate this, we examined the long-term effects of inhibiting autophagy in preimplantation embryos using CQ.

## 2 Materials and methods

### 2.1 Animals

ICR strain female and male mice, aged 8–12 weeks, were purchased from Shizuoka Laboratory Animal Center (SLC) Inc. (Hamamatsu, Japan). The mice were maintained in a SPF room (25°C, a relative humidity of 50%, and a 14/10-h light-dark cycle). Mice were fed ab libitum with a standard pelleted diet and allowed free access to distilled water. All the animal experiments were approved by the Animal Experimentation Committee at the University of Yamanashi, Japan, and conducted in accordance with the ethical guidelines.

### 2.2 Recovery and culture of 2-cell stage embryos

Female mice of the ICR strain were injected intraperitoneally with 7.5 IU of PMSG and 48 h later with 7.5 IU of hCG (ASKA Pharmaceutical, Tokyo, Japan) intraperitoneally and subjected to oestrus treatment. hCG was injected and mated with male mice of the ICR strain, and about 16 h later, a plug check was performed, Female mice with plugs were left for approximately 30 h. The mice were then euthanized by cervical dislocation, and a cut was made in the abdomen of the mice using a pair of sharp and blunt surgical scissors, tearing the skin above and below the abdomen to expose the peritoneum. The peritoneum was incised with a pair of sharp and blunt surgical scissors to remove the ovaries, fallopian tubes, and uterus of the mice, and the fallopian tubes were collected and collected in an Eppendorf tube. The collected oviducts were placed in Chatot-Ziomek-Bavister (CZB) medium, and a perfusion needle was inserted from the ovarian side while viewing under a stereomicroscope, HEPES-CZB medium (Sigma-Aldrich Chemical Co., St. Louis, MO, United States) (Kimura and Yanagimachi, 1995) was poured in and perfusion was performed, and 2-cell stage embryos were poured out from the uterine side. The emerged 2-cell stage embryos were collected with a mouse pipette, transferred between drops made of CZB medium, and the embryos were washed. Embryos were incubated in an atmosphere of 5% CO2 in air at 37°C inside an incubator until the desired developmental stage. The procedure was like previous reports with slight modifications (Hayashi et al., 2020).

### 2.3 CQ treatment

CQ was dissolved in DMSO to 5 mM and dispensed in 1 µL portions. CQ was diluted 12,500-fold, 25,000-fold, and 50,000-fold in CZB medium and used as 4 μM, 2 μM, and 1 μM, respectively. The collected embryos were cultured in droplets of CQ medium at 37°C in a humidified atmosphere containing 5% CO_2_ until use.

### 2.4 Immunofluorescence analysis

Immunostaining was performed as previously described with slight modifications (Fulka and Langerova, 2014). To further determine embryo quality, cell lineage differentiation in blastocyst stage of cultured embryos with or without supplementation of CQ were subjected to caudal type homeobox2 (Cdx2)- and Nanog-antibody immunostaining, which are markers of the inner cell mass (ICM) that will differentiate into the future fetus and trophectoderm (TE) into the placenta. In brief, embryos were washed twice in phosphate-buffered saline (PBS) containing 1% polyvinylalcohol (PBS-PVA) and fixed in 4% paraformaldehyde (PFA) in PBS for30 min at room temperature. Subsequently, embryos were washed in PBS-PVA and incubated overnight at 4°C with blocking buffer (0.1 %Triton X-100% and 1% bovine serum albumin (BSA) in PBS). Embryos were incubated overnight at 4°C in a refrigerator with the primary antibody diluted in a blocking buffer. The primary antibodies used were anti-Nanog (1:500, Abcam, ab80892) and anti-Cdx2 (1:500, BioGenex, MU392-UC). After washing in PBS-PVA, embryos were subsequently incubated with secondary antibodies, Alexa Flour 488-conjugates anti-rabbit IgG (1:500) and Alexa Flour anti-mouse IgG (1:500), for 2 h at room temperature. After washing with PBS solution, embryos were mounted on glass slides in Vecta-shield (Vector Laboratories Inc., Burlingame, CA) supplemented with 1 μg/mL 4′,6-diamidino-2 phenylindole (DAPI). The fluorescence signals were observed using a fluorescence microscope (BZ-800; Keyence, Osaka, Japan) with consistent laser settings.

### 2.5 Embryo transfer

To observe whether the addition of chloroquine to pre-implantation assisted reproductive embryos has any potential lasting effects on postnatal development, embryos were cultured, implanted into the oviducts of pseudopregnant mice, and the offspring were recovered by natural delivery. The same procedure was performed based on a previous report (Inoue et al., 2020) with some modifications. Embryos were cultured *in vitro* as described above according to 0 µM CQ, 1.0 µM CQ, and 2.0 µM CQ experimental groups. Embryos from each culture were implanted in the oviducts of pseudopregnant female ICR mice 0.5 days (dpc) after mating with vasectomized male ICR mice during the budding phase. Eight to ten embryos were transferred into each oviduct, and the embryo transfer procedure was repeated at least four times (Table 1). The pups were weighed at 19.5 dpc. In addition, naturally mated mice (NM) were used as controls. To compare the effects of *in vitro* culture with those of *in vitro* culture, we also included data from naturally mated mice (NM) as controls. For long-term observation, the same treatment was performed, and fetuses were obtained at 19.5 dpc.

**TABLE 1:** Number of embryos transferred and offspring resulting after chloroquine treatment.

CQ (µM)	No. of embryos transferred	No. host females	No. offsprings	Birth rate (%)	Birth weight ± SEM (g)
0	94	7	32	35	2.44 ±0.05 ^a^
1.0	58	4	21	37	2.43 ±0.04 ^a^
2.0	86	6	45	51	2.08 ±0.03 ^b^
Natural mating	-	-	20	-	2.03 ±002 ^b^

^a^ Values differ significantly (P < 0.05).

^b^ Values differ significantly (P < 0.05).

### 2.6 Oral glucose tolerance test (OGTT)

To determine the effect of low-concentration chloroquine treatment on blood glucose levels, weekly body weight monitoring, blood pressure measurements, and oral glucose tolerance tests (OGTT) at 8 and 16 weeks were performed. Mice were fed a normal diet and data were also compared to naturally mated mice NM. Mice from each experimental group, 0 µM CQ, 1.0 µM CQ, 2.0 µM CQ and natural crosses, at 8 or 16 weeks of age were subjected to an OGTT test with a fasting period of 6 h and no water removal. Fasting glucose was first measured, and 0.1 mL of 20% glucose per 10 g of body weight was administered orally. Blood glucose levels were measured at 15-, 30-, 60-, and 120-min intervals. The glucose tolerance graph was plotted and the area under the curve (iAUC) was calculated using the same procedure as previously reported (Ishiyama et al., 2021).

### 2.7 Systolic blood pressure observation

Eight-week-old or 16-week-old 0 µM CQ, 1.0 µM CQ, 2.0 µM CQ, and naturally mated group mice were randomly selected from each experimental group to have their blood pressure measured using the MK-2000ST NP-NIBP monitor. The blood pressure monitor was measured with the mice restrained and using a tail cuff. To reduce errors, systolic blood pressure measurements were taken at least three times. The number of mice in each group followed the number of mice obtained from embryo transfer.

### 2.8 DAPGreen staining

To examine the changes in autophagy activity induced by low-concentration chloroquine treatment, DAPGreen-Autophagy Detection (DAPGreen) staining was performed on chloroquine-treated embryos, untreated and naturally mated mulberry embryos at the morula embryo stage. The embryos were dissolved in DMSO and incubated in CZB medium diluted 200-fold with DAPGreen prepared 0.02 mM and aliquoted to 1 µM for 30 min in an incubator, then the embryos were washed with CZB medium and incubated again for 30 min in an incubator. The fluorescence signals were observed using a fluorescence microscope (BZ-800; Keyence, Osaka, Japan) with consistent laser settings.

### 2.9 Statistical analysis

Statistical analyses were conducted using JMP Pro software version 17.0 (SAS Institute Inc., Cary, NC). Data were analyzed using one-way ANOVA followed by Tukey’s or Fisher’s LSD multiple comparison tests when appropriate. P-values less than 0.05 were categorized as statistically significant. Graph figures were generated by GraphPad Prism 10 Version 10.3.1 (2024). Logistic regression analysis was also used in this experiment to examine these relationships, with the presence or absence of 2.0 μM CQ in the CZB as the dependent variable and the number of litters per mother and litter weight as independent variables.

## 3 Results

### 3.1 Preimplantation development under low-concentration CQ treatment

**FIGURE 1:**
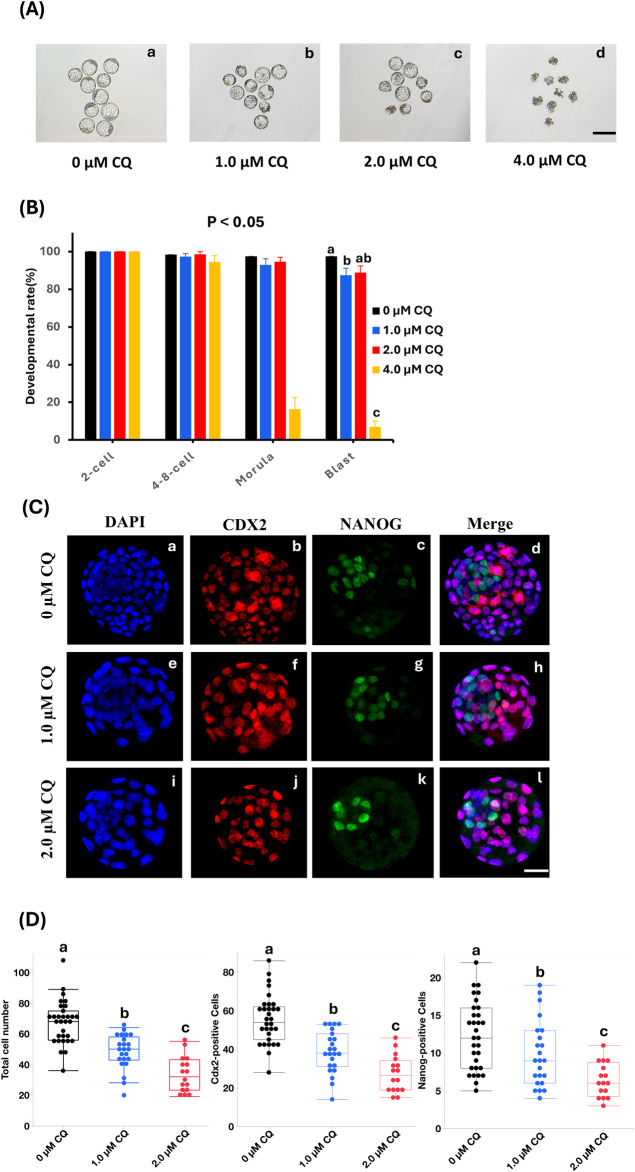
Effects of low-concentration chloroquine treatment on development and cell proliferation. **(A)** Representative images of IVV blastocysts cultured in CQ-supplemented medium at 1.0 µM, 2.0 µM, and 4.0 µM in low-concentration CQ-treated sections. Scale bar = 100 µm. 0 μM CQ treatment. **(B)** Incidence to blastocyst stage in 4 groups: 0 µM CQ, 1.0 µM CQ, 2.0 µM CQ, and 4.0 µM CQ after 2-cell stage perfusion. P < 0.05. 0 μM CQ treatment (a) 1.0 µM CQ treatment (b) 2.0 µM CQ treatment (c) 4.0 µM CQ treatment (d). **(C)** Next, representative images of NANOG (green), CDX2 (red) and DAPI (blue) immunostaining of IVV blastocysts in the three groups of 0 µM CQ, 1.0 µM CQ and 2.0 µM CQ. Scale bar = 25 µm. DAPI, CDX2, NANOG, and Merge immunostaining images of 0 µM CQ treatment zone (a–d); immunostaining images of 1.0 µM CQ treatment zone (e–h); immunostaining images of 2.0 µM CQ treatment zone (i-l). **(D)** Comparison between 3 groups after measuring total cell count, CDX2, and NANOG positive cell count. Data represent means ± SEM for each treatment group obtained from at least three independent replicates. Different letters represent significant differences (P < 0.05).

To assess the concentration dependence of CQ treatment during preimplantation development, embryos were cultured and observed in media containing 1.0 µM, 2.0 µM, or 4.0 µM CQ. We have previously evaluated the effects of CQ treatment on preimplantation embryo development (Uechi et al., 2025). We started with high concentrations of CQ and found that 4 µM CQ treatment resulted in developmental arrest in blastocysts. On the other hand, we found that 2 µM CQ was the lowest concentration that allowed embryo development when we decreased the concentration step by step to examine embryos for implantation. Embryos in the CQ-treated groups were exposed to CQ from the 2-cell stage for 48 h, then cultured in CZB medium until the blastocyst stage. As shown in Figures 1A,B, the blastocyst development rate decreased in a concentration-dependent manner following CQ treatment. In particular, the blastocyst formation rate remained above 80% for treatments up to 2.0 µM CQ (Figure 1A, a–c). However, at 4.0 µM CQ, blastocyst development was markedly reduced (Figure 1A, d). Next, to clarify the effects of low CQ concentrations on embryonic development, immunostaining was performed using Cdx2 and Nanog antibodies, which are molecular markers for the trophectoderm (TE) and inner cell mass (ICM), respectively (Figure 1C). Compared to the untreated group, both 1.0 µM and 2.0 µM CQ treatments significantly reduced the total number of cells, as well as the number of Cdx2-positive and Nanog-positive cells (Figure 1D). Moreover, a CQ concentration-dependent decrease in cell numbers was observed (Figure 1D).

### 3.2 Visualization of autophagy activity changes by DAPGreen staining

**FIGURE 2:**
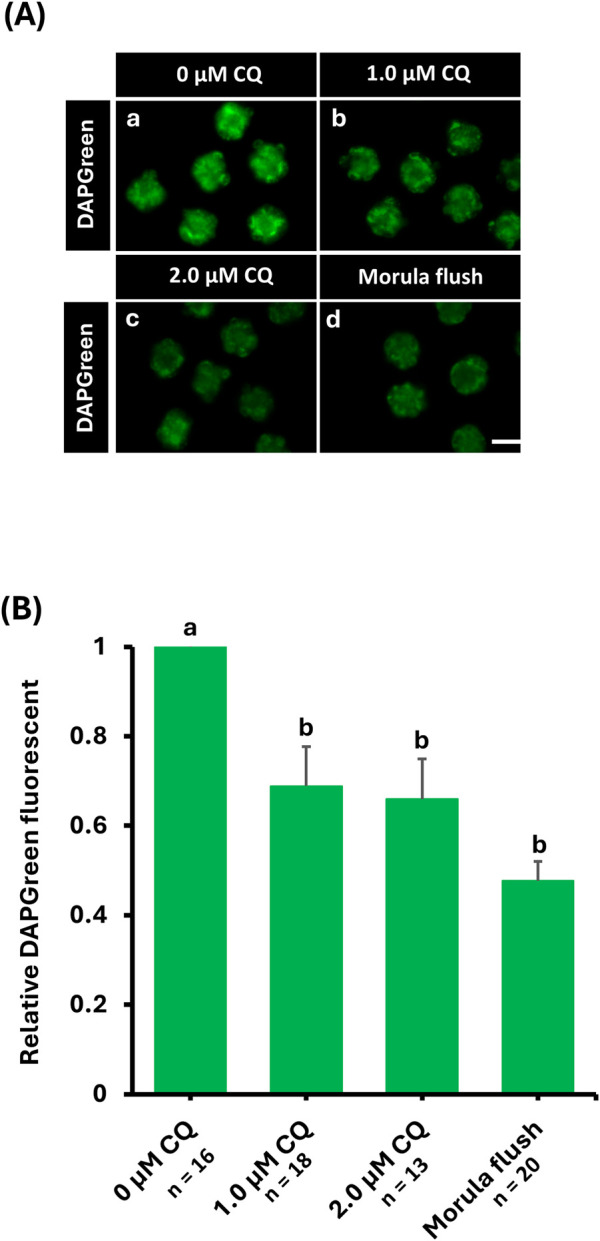
**(A)** Relative fluorescence intensity or representative images of autophagy activity by *in vitro* culture and chloroquine treatment. Autophagy activity was evaluated by DAPGreen staining or DAPGreen stained images after *in vitro* culture to morula embryo stage. 0 μM CQ (a); 1.0 µM CQ (b); 2.0 µM CQ (c); morula embryo stage. Embryos obtained by perfusion manipulation after *in vitro* culture until the mulberry embryo stage (d). Scale bar = 10 µm. **(B)** DAPGreen relative fluorescence intensity when the untreated area is set as 1. Data represent means ± SEM for each treatment group obtained from at least three independent replicates. Different letters represent significant differences (P < 0.05).

DAPGreen is a fluorescent probe used to detect autophagy, emitting fluorescence upon incorporation into newly formed autophagosomes (Iwashita et al., 2018). To compare changes in autophagy activity caused by *in vitro* culture and CQ treatment, DAPGreen staining was performed on morula-stage embryos from the untreated, 1.0 µM CQ-treated, 2.0 µM CQ-treated, and in vivo-fertilized groups (Figure 2A). Fluorescence intensities were normalized to the average intensity of the untreated group, set as 1, and the relative DAPGreen fluorescence of CQ-treated and in vivo-fertilized embryos was compared accordingly. Moreover, the higher intensity of DAPGreen fluorescence was observed in the untreated group (Figure2A, a). In contrast, CQ treatment significantly reduced and altered the localization of fluorescence intensity (Figure2A, b-d). There were significant differences between the untreated group and the 1.0 µM CQ-treated (0.69 ± 0.09), 2.0 µM CQ-treated (0.66 ± 0.09), and *in vivo* embryos (0.48 ± 0.04) (P < 0.05). However, no significant differences were observed among the 1.0 µM CQ-treated, 2.0 µM CQ-treated, and *in vivo* groups (Figure 2B). In summary, CQ treatment tended to reduce autophagy activity at the morula stage. The levels also tended to approach those of *in vivo* embryos, which exhibited the lowest autophagy activity.

### 3.3 Effects of low-concentration CQ treatment on postnatal development

**FIGURE 3:**
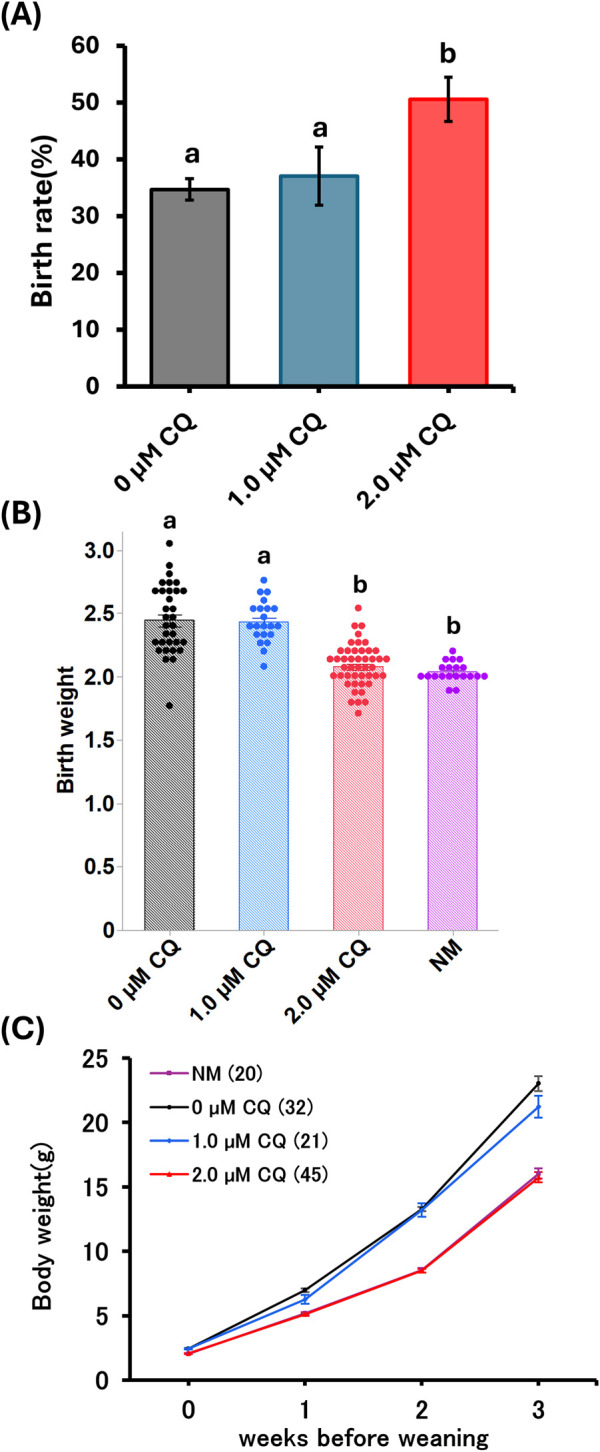
Postnatal phenotype after low chloroquine treatment. **(A)** Comparison of pup’s rate born by low-concentration chloroquine treatment. **(B)** Pup weights obtained by spontaneous parturition at 19.5 dpc from the natural mating and experimental groups. **(C)** Weaning weight measurements of mice from week 0 to week 3. Data represent means ± SEM. The number of offspring per treatment group is noted in the legend. The data were obtained from at least three independent experimental replicates. Abbreviations: NM, natural mating.

As shown in Table 1, comparisons were made on pups obtained after embryo transfer of preimplantation embryos treated with low concentrations of CQ. Comparison of offspring rates was made for pups obtained by embryo transfer following *in vitro* culture with low-concentration CQ treatment (Figure 3A). No significant differences were observed between the 1.0 µM CQ treatment (37% ± 5.1) and the untreated group (35% ± 1.9). However, the offspring rate in the 2.0 µM CQ treatment (51% ± 3.9) was significantly higher than in the untreated group (P < 0.05). Figure 3B shows a comparison of offspring weights across treatments. The pups from *in vitro* culture weighed the most, averaging 2.44 g. These results are consistent with previous studies demonstrating the effects of *in vitro* culture and culture media on birth weight. There was no significant difference in birth weight between the untreated group and the 1.0 µM CQ treatment group, which had an average birth weight of 2.43 g. However, significant differences were observed between the untreated group and the 2.0 µM CQ treatment group, as well as between the 1.0 µM and 2.0 µM CQ treatment groups. CQ treatment tended to reduce body weight compared to untreated groups. Interestingly, the 2.0 µM CQ treatment group, with an average birth weight of 2.08 g, had weights comparable to the natural mating group, which averaged 2.03 g. In this experiment, since the number of offspring per mother is suggested to affect offspring weight, logistic regression analysis was conducted using both the number of offspring per mother and offspring weight as explanatory variables, with 2.0 µM CQ treatment (yes/no) as the dependent variable. In this analysis, the 2.0 µM CQ treatment group was designated as the event group. The results showed an odds ratio of 0.018 for offspring weight (P = 0.0003), which is less than 1 and indicates a significant association. Since the odds ratio for pup weight was below 1, it is statistically likely that 2.0 µM CQ treatment contributes to a reduction in pup weight, independent of the number of pups per mother (Figure 3B; Table 2). One week after birth (Figure 3C), we observed that the weaning weights of the pups fell into two distinct groups: the untreated and 1.0 µM CQ-treated groups showed higher weight gain, while the naturally mated and 2.0 µM CQ-treated groups exhibited moderate weight gain. Notably, the weaning weights of the 2.0 µM CQ-treated pups closely resembled those of the naturally mated group.

**TABLE 2:** Logistic regression analysis of litter size and pups weight with 2.0 µM CQ treatment.

Independent variable	Odds ratio	Lower limit of 95% confidence interval	95% confidence interval upper limit	P Value
No. of offspring per mother	1.201	0.989	1.460	0.0641
Birth weight	0.018	0.002	0.192	0.0003

### 3.4 Effects of low-concentration CQ treatment on long-term weight change and metabolism

**FIGURE 4:**
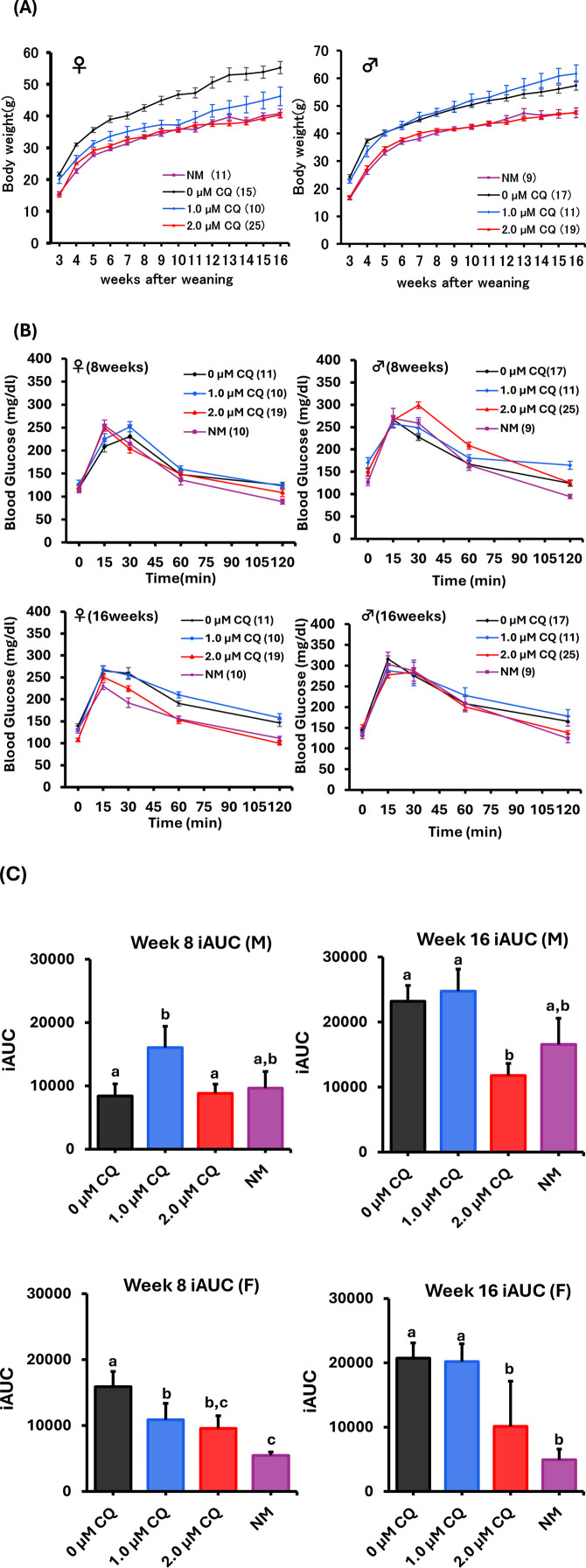
Weight change and Oral Glucose Tolerance Test (OGTT) study up to 16 weeks with low-concentration CQ treatment. **(A)** Body weight of females and males from post-weaning to 16 weeks. Each experimental plot is 0 µM CQ, 1.0 µM CQ, and 2.0 µM CQ, including natural mating. Data represent means ± SEM. The number of offspring per treatment group is noted in the legend. The data were obtained from at least three independent experimental replicates. **(B)** Females (8 and 16 weeks old) and males (8 and 16 weeks old). Glucose tolerance test graphs. **(C)** Females (8 and 16 weeks old) and males (8 and 16 weeks old). Area under the curve. Data represent mean ± SEM. The number of offspring per treatment group, obtained from at least three independent experimental replicates, is noted in the legend. Abbreviations: NM, natural mating.

Weight changes from post-weaning to 16 weeks were examined to evaluate the long-term effects of low-concentration chloroquine treatment on preimplantation embryos (Figure 4A). In females, the highest growth curve was observed in the untreated group, while the low-concentration CQ treatments resulted in lower body weights. Notably, the 2.0 µM CQ treatment group showed a growth curve nearly identical to that of the naturally mated group, with the lowest body weight at 16 weeks among all experimental groups. In males, there was a pronounced difference in body weight between the untreated and naturally mated groups. Interestingly, at 16 weeks, the 1.0 µM CQ treatment group had the highest body weight. However, both males and females in the 2.0 µM CQ treatment group displayed growth patterns similar to those of the naturally mated group (Figure 4B). Oral glucose tolerance tests (OGTT) were performed at 8 and 16 weeks to compare metabolic function among untreated, 1.0 µM CQ-treated, 2.0 µM CQ-treated, and naturally mated groups. One-way analysis of variance (ANOVA) was primarily used in this study to assess significant differences between experimental groups (Figure 4C). The incremental area under the curve (iAUC) from the OGTT was calculated, revealing the highest values in males treated with 1.0 µM CQ. Conversely, at 16 weeks of age, the 2.0 µM CQ treatment group exhibited the lowest iAUC, which was significantly different from the untreated group. In females, the highest iAUC values were observed in the untreated group at both 8 and 16 weeks, while the lowest values were seen in the naturally mated group. These differences were statistically significant, as were those between the untreated and 2.0 µM CQ treatment groups. In summary, at 16 weeks, significant differences were found between the untreated and 2.0 µM CQ treatments for both sexes, with the 2.0 µM CQ group showing values closer to those of the naturally mated group (P < 0.05).

## 4 Discussion

In this study, we focused on the autophagy pathway, which plays a crucial role in nutrient supply during early embryogenesis and investigated the effects of inhibiting autophagy activity during preimplantation embryo culture using CQ on subsequent offspring and adults. We found that after implantation, the 2.0 µM CQ-treated group exhibited increased pups rate and reduced body weight comparable to the naturally mated group, and glucose tolerance like that of the naturally mated group, in contrasted to the untreated group. Our findings suggest that inhibiting autophagy could reduce the long-term risks associated with *in vitro* embryo culture. To our knowledge, this is the first study to demonstrate a link between autophagy during preimplantation development and long-term outcomes.

Blastocyst development rates were 88% in 1.0 µM CQ, 89% in 2.0 µM CQ, and only 6.9% in the 4.0 µM CQ, compared to 97% in the untreated control. The 4.0 µM CQ treatment caused a significant developmental arrest between the 4-8 cell stage and the morula stage. These findings are consistent with previous studies indicating that autophagic activity is essential for mammalian preimplantation development beyond the 4- to 8-cell stage (Tsukamoto et al., 2008b). Evaluation of total cell count, Cdx2-positive cell count, and Nanog-positive cell count revealed a significant concentration-dependent decrease in all parameters with increasing CQ concentrations. This observation raises the possibility that autophagy is required not only for embryonic development but also for cell proliferation beyond the 4-8 cell stage.

To investigate changes in autophagy activity induced by low-concentration CQ treatment, we performed DAPGreen assay. The results showed that autophagy activity decreased in the 1.0 µM and 2.0 µM CQ treatments compared to the untreated controls. Unexpectedly but consistently, lower autophagy activity was also observed in morula–stage embryo flushed from the oviducts–i.e., *in vivo* fertilized and developed embryos-indicating a physiological baseline level of autophagy at this stage. These results indicate that autophagy activity is higher in embryos cultured *in vitro* than in those fertilized and developed *in vivo*. No significant differences were observed between the 1.0 µM CQ and 2.0 µM CQ treatment groups. CQ inhibits autophagy by blocking the fusion of autophagosomes with lysosomes, thereby preventing autophagic degradation (Mauthe et al., 2018). DAPGreen is a fluorescent probe that labels autophagosomes during their formation, enabling real-time visualization of autophagic activity in live cells (Iwashita et al., 2018). Therefore, the observed reduction in DAPGreen signal suggests that CQ treatment not only blocked autophagosome degradation but also suppressed autophagosome formation. CQ is known to block autophagic flux by preventing the fusion of autophagosomes with lysosomes (Mauthe et al., 2018). However, the mechanism by which CQ treatment downregulates *de novo* autophagosome formation remains unclear. Taken together, these findings suggest that the *in vitro* culture environment elevates autophagy activity, potentially leading to altered metabolism and long-term outcomes. Nevertheless, it remains unknown whether suppressing autophagic degradation itself or reducing overall autophagy activity is more effective in mitigating these long-term effects.

Autophagy is known to respond dynamically to the cellular environment. For example, nutrient starvation induces autophagy in eukaryotic cells primarily through inhibition of the target of rapamycin (TOR) pathway (Jung et al., 2010). In embryos, autophagy is also influenced by environmental factors such as glucose concentration (Adastra et al., 2011). Although the exact mechanism underlying elevated autophagy in in vitro–cultured embryos remains unclear, IVF-generated blastocysts exhibit increased reactive oxygen species (ROS) and oxidative damage (Lee et al., 2022), both of which are known to upregulate autophagy (Kroemer et al., 2010).

In order to clarify the effects of CQ treatment on long-term outcomes, we examined changes in pup rate, pup weight, blood glucose, and blood pressure in offspring derived from morula-stage embryos. Surprisingly, pups weights were like those of the naturally mated groups. The untreated group had the highest body weight. The 2.0 µM CQ treatment was like the natural mating group until 16 weeks post-weaning for both sexes. The untreated group remained heavier than the naturally mated group. The blood glucose levels focused on the 8-week and 16-week age groups. In male mice, there was no significant difference between the untreated and CQ-treated groups at 8 weeks of age, but at 16 weeks of age, the iAUC value was the lowest in the 2.0 µM CQ treatment group and significantly different from the untreated group. On the other hand, there was no significant difference between the untreated and 1.0 µM CQ -treated groups. For female mice, significant differences were observed between the untreated and 2.0 µM CQ -treated groups at 8 weeks of age. The difference was even more pronounced at 16 weeks of age. However, there was no significant difference between the untreated and 1.0 µM CQ treatments. Thus, these results suggest that the 2.0 µM CQ treatment tended to be closer to the natural mating group’s glucose metabolism in both males and females and may suppress the metabolic abnormalities in the untreated group.

Based on the significant improvements in pup rate and pup weight observed in the 2.0 µM CQ treatment group, we conclude that 2.0 µM represents the optimal low concentration of CQ required to suppress the long-term adverse effects of *in vitro* culture. How does CQ treatment during preimplantation development suppress long-term outcomes? At present, it remains unclear whether CQ exerts this effect directly or indirectly. If the inhibition or suppression of autophagy by CQ is directly involved in mitigating long-term outcomes, it can be speculated that excessive autophagic degradation impairs cellular functions (Sandri, 2010), thereby altering metabolic programming (Lee et al., 2022) and leading to long-term consequences. Interestingly, although autophagy activity during preimplantation development showed no significant difference between the 1.0 µM and 2.0 µM CQ treatment groups, the 2.0 µM CQ treatment more effectively suppressed postnatal phenotypes. This suggests that inhibiting autophagic degradation may play a more critical role in mitigating long-term effects than merely suppressing overall autophagy activity.

Currently, there are few successful reports of mitigating the long-term effects associated with embryo culture. One study reported that melatonin supplementation in the culture medium rescued impaired glucose metabolism in IVF mouse offspring (Jia et al., 2022). Although the exact mechanism remains unclear, melatonin’s beneficial effects are speculated to involve multiple pathways, including protection of mitochondrial function (Jia et al., 2022). Interestingly, melatonin has also been shown to significantly inhibit autophagy in certain *in vivo* and *in vitro* contexts (Li et al., 2022; Kang et al., 2014). Moreover, melatonin reduces Zearalenone-induced autophagy in porcine embryos (Xu et al., 2019). Therefore, our findings suggest that the beneficial effects of melatonin in culture media may mitigate long-term outcomes partially through the suppression of autophagy.

In addition, a number of large meta-analyses and population cohort studies oin human *in vitro* culture and ART show in the literature various but generally modest differences in fasting glucose/insulin, lipid profiles, blood pressure, and adiposity between ART and naturally conceived (NC) children, many of which decrease with age (Clark, 1992; Yeung et al., 2013; Cui et al., 2020; Huang et al., 2021; Pinborg et al., 2023). The results of the present experiments are consistent with findings in the literature. On the other hand, the phenotypic differences between mouse models and humans still require further investigation. It is also possible that the difference in weight between the IVC untreated group and the NM group contributes to the discrepancy between the present mouse study and the clinical findings. Such a difference has not been observed in humans, at least in cases involving fresh embryo transfer. Studies have shown that infants conceived via IVF have lower birth weights and a higher incidence of birth defects compared to those conceived spontaneously (Behr and Wang, 2004). These issues may be attributable to differences in the composition of the culture media used for mouse and human embryos. Although it is difficult to identify the specific components responsible for the difference, the levels of amino acids (AAs), including the branched-chain AAs leucine, isoleucine, and valine, to which embryos are exposed, are suggested to influence blastocyst development and long-term effects (Eckert et al., 2012). Therefore, differences in amino acid (AA) composition in the culture media could contribute to the observed outcomes, as the CZB medium used in this study contains no AAs, whereas the medium for human embryos does (Chatot et al., 1989; Zagers et al., 2025). Further, since this experiment was conducted using *in vivo* fertilized embryos rather than IVF embryos, the underlying causes of these issues require further investigation.

We used in vivo-fertilized embryos cultured *in vitro* as a model, since embryo culture is the major factor contributing to placental abnormalities (Vrooman et al., 2020), which may be in turn linked to long-term effects (Burton et al., 2016). Future study should investigate the effects of CQ treatment on IVF embryos, their long-term outcomes and the underlying mechanisms. Future study should investigate the effects of CQ treatment on IVF embryos, their long-term outcomes and the underlying mechanisms.

## 5 Conclusion

This study suggests that altered autophagy activity contributes to adverse long-term outcomes. According to the DOHaD theory, the risk of developing lifestyle-related diseases such as diabetes and hypertension is strongly influenced by the nutritional and health environment during the fetal period and early postnatal life (Langley-Evans and McMullen, 2010; Aiken and Ozanne, 2014). Independent of developmental stage, nutrient deficiency can increase autophagy (Yang et al., 2020), which may elevate disease risk through a shared underlying mechanism. Thus, our study provides new insight into the mechanisms underlying the DOHaD theory. On the other hand, this study is limited to mouse models, and its direct applicability to humans and clinical IVF outcomes remains speculative. Therefore, further investigation is needed to explore the potential clinical application of CQ.

## Data Availability

The original contributions presented in the study are included in the article/supplementary material, further inquiries can be directed to the corresponding author.
